# Upregulation of Copine1 in trabecular meshwork cells of POAG patients: a membrane proteomics approach

**Published:** 2008-05-30

**Authors:** Yuehong Zhang, Qianying Gao, Shan Duan, Yuan He, Xuerong Sun, Ruzhang Jiang, Yongheng Duan, Xiufeng Zhong, Jian Ge

**Affiliations:** Zhongshan Ophthalmic Center, Sun Yat-sen University, Guangzhou, China

## Abstract

**Purpose:**

Primary open-angle glaucoma (POAG) is a leading cause of irreversible blindness worldwide, and its pathogenesis is still unknown. The purpose of this study was to determine molecular changes in membrane proteins in trabecular meshwork (TM) cells from POAG patients compared to those of age-matched normal controls.

**Methods:**

Two-dimensional (2-D) gel electrophoresis profiles of membrane extracts from normal and glaucomatous TM cells were compared. The desired spots were identified after trypsin digestion and mass spectrometric analysis. Based on the results, a calcium-dependant membrane-binding protein, copine1, was further approached for a possible role in glaucomatous TM cells. The intracellular calcium concentration ([Ca^2+^]i) of TM cells was increased by incubating with calcium ionophore, A23187. Relative quantification real-time polymerase chain reaction (PCR) and western blot analysis measured copine1 expression and localization both in untreated and A23187-treated TM cells.

**Results:**

Real-time PCR and western blot analysis confirmed that *copine1* mRNA and protein expression were upregulated in glaucomatous TM cells when compared to normal ones. The cell distribution studies further showed that copine1 existed both in the membrane and cytoplasm fractions of glaucomatous TM cells but existed exclusively in cytoplasm fractions of their normal counterparts. More importantly, an influx of Ca^2+^ markedly promoted the translocation of copine1 from the cytoplasm to membranes in glaucomatous TM cells.

**Conclusions:**

Copine1 is upregulated in plasma membranes of TM cells in individuals with POAG, which may be partly explained by its Ca^2+^-dependent translocation from the cytoplasm to the membranes. Investigation of the role and functions of copine1 in TM cells should offer new insight into the abnormal intracellular Ca^2+^-signaling pathway in glaucomatous TM and help to clarify the molecular mechanism of POAG.

## Introduction

Glaucoma is the term used to describe a group of irreversible blinding diseases characterized by progressive optic neuropathy. Primary open-angle glaucoma (POAG) is the most common form of this disease, and its pathogenesis is still unknown [1]. It is widely accepted that elevated intraocular pressure (IOP), which is determined by the relative rates of inflow and outflow of aqueous humor, is a consistent risk factor associated with the development and progression of POAG [2,3]. As a major site of outflow resistance to the aqueous humor, the trabecular meshwork (TM) plays an important role in regulating IOP. Therefore, cultured TM cell models are indispensable study objects in the field of glaucoma [4,5].

Like those of any cell, the pathological features of TM cells are determined largely by molecular changes in protein expression [4-6]. Identification of differential proteins from TM cells of patients with POAG and those from healthy individuals in the same age range would help to understand the pathogenesis of POAG. Proteomics is an excellent way to screen for alterations in protein expression and has proved helpful in studying the pathogenesis of POAG [4,5,7,8]. Characterizing membrane proteomes using large-scale, high-throughput approaches helps significantly in understanding the role of membrane proteins in physiopathology processes and drug discovery. To our knowledge, however, few TM proteome studies focus mainly on soluble protein expression [4,5,8,9], and membrane proteomes of TM cells are little understood at present.

In this study, we initiated a comparative proteomic survey of TM cell membrane extracts from normal and POAG individuals and verified the upregulation of copine1 in TM cell membranes of POAG patients. A growing body of evidence suggests that copine1 may mediate an array of cellular processes by conferring Ca^2+^ regulation to various signaling pathways [10-13]. We elucidated further the ability of Ca^2+^, introduced into the cytoplasm of TM cells by applying calcium ionophore, A23187, to markedly promote the translocation of copine1 to POAG patient’s TM cell membrane fractions.

## Methods

### Tissue procurement and cell culture

Normal eyes from five human donors were obtained from the Zhongshan Ophthalmic Center Eye Bank within 24 h of death (due to automobile accidents). The ages of the donors ranged from 20 to 60 years. According to the records, no known eye diseases were detected in these samples used in the present study. After enucleation, all eyes were cut equatorially behind the ora serrata. The ciliary body, iris, and lens were removed, and the TM was dissected under a microscope (40X). After obtaining written informed consent, TM specimens from five POAG patients (15–60 years old) were acquired by standard surgical trabeculectomy for therapeutic purposes less than 1 h after surgery. Prior to the surgery, clinical data was collected on each patient including age, gender, use of prostaglandin analogs, number of argon laser trabeculoplasty and other ocular surgical interventions, type and duration of glaucoma, IOP, and visual acuity. Glaucoma diagnosis was based on careful clinical eye examination including slit lamp, optical coherence topography, gonioscopy, fundus-photography, and visual field. The TM tissues from POAG (GTM) and age-matched non-diseased (NTM) individuals were used to generate independent primary cultures of TM cells as previously described [14]. The samples were not pooled at any time in these experiments. Primary cultures were used at passages 4 and 5 for each experiment. Cells were grown in Dulbecco’s modified Eagle’s medium (DMEM; Gibco, Grand Island, NY) supplemented with 2 mM glutamine, 50 units/ml penicillin, 0.1 mg/ml streptomycin, and 10% fetal bovine serum. Cells were maintained in a 37 °C humidified incubator in the presence of 5% CO_2_. Phase contrast microscopy confirmed that confluent cultures formed a monolayer with the typical morphological characteristics of cultured human TM cells.

### Immunocytochemistry

The immunocytochemical detection of TM specific antibodies was performed as previously described [15-17]. Briefly, TM cells were seeded onto polylysine (10 μg/ml)-coated glass chamber slides at a density of 2,000 cells per chamber. After rinsing the cultures with PBS, the cells were fixed in ice-cold 4% paraformaldehyde for 15 min and treated for 4 min in 100 mM phosphate buffer, 1 mg/ml bovine serum albumin (BSA), and 0.2% Triton X-100 to permeabilize the cell membranes. After quenching the endogenous peroxidase activity with 3% H_2_O_2_, the cells were incubated with 0.5% blocking reagent for 30 min (TSA-Direct kit, Dupont-NEN, Boston, MA) and then immunolabeled with one of the following antibodies at room temperature for 1 h: mouse monoclonal anti-fibronectin (1:200), rabbit polyclonal anti-laminin (1:200), mouse monoclonal anti-vimentin (1:200), mouse monoclonal anti-neuron specific enolase (1:200), and mouse monoclonal anti-Factor VIII (1:200). After incubation with the primary antibody, the cells were rinsed with PBS and incubated for an additional 45 min with biotinylated goat anti-mouse IgG (1:300; Vector Laboratories, Burlingame, CA) or anti-rabbit IgG (1:300, Vector Laboratories) where appropriate followed by avidin-biotin-peroxidase complex for 10 min. After a series of washes, the specimens were treated with 3,3′-diaminobenzidine (DAB)/peroxidase reaction (Vector DAB substrate kit; Vector Laboratories), washed in water, treated with hematoxylin counterstain, washed again, and then dried at room temperature. The samples were then dehydrated in a graded series of alcohols and coverslipped with DPX. Phase contrast microscopy confirmed the staining pattern for each antibody.

### Sample preparation

Crude membrane and cytoplasm extracts were prepared as previously described with minor modifications [18,19]. Briefly, TM cells were washed twice with PBS, scraped from the culture flask, and centrifuged at 1,500x g for 5 min at 4 °C. The cells were then resuspended in a calcium-free lysis buffer (50 mM Tris/HCl, pH 7.4, 1% [v/v] Triton X-100, 1% [v/v] protease inhibitor cocktail), frozen in liquid nitrogen, and thawed at room temperature. They were then centrifuged at 200,000x g for 45 min at 4 °C to obtain the supernatant as a cytoplasmic fraction. After the pellets were washed with the ice-cold lysis buffer, they were resuspended in 4 ml of ice-cold 100 mM Tris/HCl (pH 7.4). The supernatant was then centrifuged at 50,000x g for 20 min at 4 °C, and the resultant membrane pellet resuspended in 100 mM Tris/HCl (pH 7.4). When treatment with calcium ionophore was required, cells were resuspended in Tris-buffer saline (TBS; 10 mM Tris/HCl, pH 7.5, 150 mM NaCl) containing 1 mM CaCl_2_, 5 μM A23187 and then incubated at 37 °C for 30 min. Following the incubation, all media were removed, and the TM cells were suspended in calcium-free lysis buffer.

### Two-dimensional electrophoresis

Identical membrane samples containing 100 µg proteins were dissolved in 300 µl of a rehydration buffer containing 7 M urea, 2 M thio-urea, 4% (w/v) CHAPS, 20 mM Tris,10 mM DTT, 0.2% (v/v) ampholytes (pH 3–10; Bio-Rad, Hercules, CA), and a trace of bromchlorphenol blue. The solution was applied to IPG strips (pH 3–10, 17 cm, Bio-Rad). IEF was performed after rehydration of the IPG strips. The gel strips were then equilibrated twice for 15 min by gently shaking them in 8 ml of an equilibration solution containing 6 M urea, 0.05 M Tris-HCl, 2% SDS, and 20% glycerol. DTT (1% w/v) was added to the first sample, and iodoacetamide (2.5% w/v) was added to the equilibration solution. SDS–PAGE was performed using 13% polyacrylamide gels. At the least, triplicate gels were run in parallel for each sample. Silver staining resulted in protein visualization, and the stained gels were scanned with an image scanner (Amersham Biosciences Ltd, New Territories, HK) and analyzed using ImageMaster 2-D Platinum software (version 5.0, Amersham Bioscience, New Territories, HK).

### Protein identification by MALDI-TOF-MS/MS

Protein spots were excised from the gels and placed into a 96 well microtiter plate. Gel pieces were isolated in a solution of 15 mM potassium ferricyanide and 50 mM sodium thiosulfate (1:1) for 20 min at room temperature. They were then washed twice with deionized water and shrunk by dehydration in acrylonitrile (ACN). The dried gel pieces were digested overnight in 12.5 ng/ml trypsin in 25 mM ammonium bicarbonate. The peptides were extracted twice by 60 μl of 50% ACN/0.1% TFA for 20 min each and dried with N_2_. Peptides were mixed with 5 mg/ml cyano-4-hydroxycinnamic acid (CHCA) matrix (Sigma–Aldrich, Steinheim, Germany) in 50% ACN/0.1% TFA, spotted onto a matrix-assisted laser desorption/ionization (MALDI) target and then analyzed by a 4700 Proteomics Analyzer (Applied Biosystems, Foster city, CA). After mass spectrometry (MS) acquisition, six of the strongest peptides per spot were selected automatically for tandem mass spectrometry (MS/MS) analysis. Data were searched using the MASCOT search engine (Matrix Science, London, UK). The mass range was scanned from 700 Da to 3200 Da. The mass tolerance was set as 0.2 Da, and MS/MS tolerance was 0.6 Da. The automatic data analysis and database search were performed using GPS Explore software (Applied Biosystem).

### Quantitative real-time polymerase chain reaction

Total RNA was isolated from the TM cells using the Trizol RNA extraction reagent (Invitrogen Corp, Guangzhou, China) according to the manufacturer’s instructions. Purified RNA was reverse-transcribed using a SYBR PrimeScriptTM RT–PCR Kit (TaKaRa Corp, Dalian, China). Real-time quantification of *copine1* mRNA was performed on an ABI PRISM 7000 Sequence Detection System using SYBR Green I as the reporter dye (TaKaRa Corp). The comparative C_t_ method was employed while the relative quantity of the target gene mRNA, normalized to *GAPDH* and relative to the calibrator, was expressed as fold change=2^-ΔΔCt^. The following human *copine1* and *GAPDH* oligonucleotide primers were respectively used in the real-time quantitative polymerase chain reaction (PCR): cpn1-F: 5′-ACC TTG GTT CAG CTG TCC ATT TC-3′; cpn1-R: 5′-AGT TCC GCA CCC GTT CAG TC-3′ and GAPDH-F: 5′-GCA CCG TCA AGG CTG AGA AC-3′; GAPDH-R: 5′-TGG TGA AGA CGC CAG TGG A-3′. Duplicate PCR reactions were tested using the following amplification protocol: 95 °C for 10 s followed by 38 cycles at 95 °C for 5 s and at 60 °C for 31 s.

### Western blot analysis

Proteins were separated using 12% SDS-polyacrylamide gels. The resolved proteins were transferred electrically to PVDF membranes and incubated with 5% skim milk in TBS with 0.05% Tween-20. The membrane was probed with a mouse monoclonal copine1 antibody (1:1000 dilution; Novus Biologicals, Guangzhou, China) at 4 °C overnight. It was then incubated with horseradish peroxidase (HRP)-conjugated anti-mouse antibody (1:5000 dilution; Sigma) for 1 h at room temperature. As an internal control, the levels of β-actin were examined at the same time. After washing, the immunoreactive bands were detected using ECL chemiluminescence reagents. The density of the bands was quantified using a laser densitometer (ATTO densitograph 4.0, Fujifilm, Tokyo, Japan). Each experiment was repeated three times. The statistical difference of the data was determined by the Student's *t*-test.

### Measurement of intracellular calcium concentration

Measurements of intracellular calcium concentration ([Ca^2+^]i) were performed using the fluorescent Ca^2+^ indicator Fura3-acetoxymethyl ester (Fura 3 AM; Sigma). TM cells (5×10^5^) were collected by trypsinization, washed in PBS, and then incubated for 30 min at 37 °C with Fura 3 AM and washed twice with PBS to remove any extracellular dye. *Cells were pelleted at 200x g for 5 min, washed in PBS, and then analyzed immediately by flow cytometry analysis using a BD FACS AriaTM flow cytometer and BD FACSDiVa software (Becton Dickinson, Mountain View, CA).* Photomultiplier settings were adjusted to detect green fluorescence (λ_em_=488 nm) of Fura 3 AM on the filter detector. In each experiment, at least 20,000 events were analyzed. The relative fluorescence intensity values were used for data presentation. The statistical difference of the data was determined by the Student's *t*-test.

## Results

### Immunohistochemical assays confirmed the primary cultures were trabecular meshwork cells

**Figure 1:**
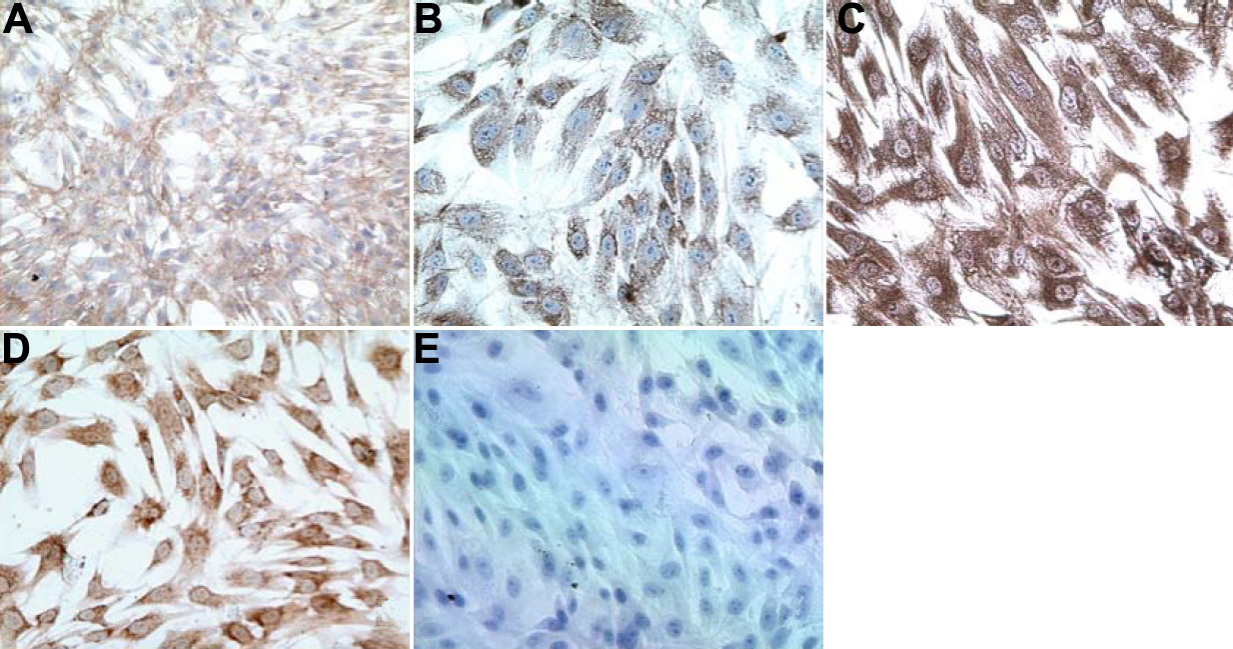
Immunohistochemical characterization of primary trabecular meshwork cell cultures. Immunohistochemical assays on the TM cells were positive for fibronectin (**A**), laminin (**B**), vimentin (**C**), neuronal specific enolase (**D**) and negative for the endothelial cell marker, factor VIII (**E**), which indicated that the primary cultures obtained from normal and POAG individuals were indeed TM cells.

Immunohistochemical characterization of primary TM cell cultures was shown in Figure 1. We confirmed that the primary cultures obtained from both normal and POAG individuals were indeed TM cells since they were immunopositive for TM specific molecular markers, extracellular matrix molecules (fibronectin and laminin), the cytoskeletal molecule, vimentin, and neuronal specific enolase. The cultures were also immunonegative for the endothelial cell marker, factor VIII, as has been reported by others [15-17].

### Membrane proteomics revealed upregulation of copine1 in GTM cell membranes

**Figure 2:**
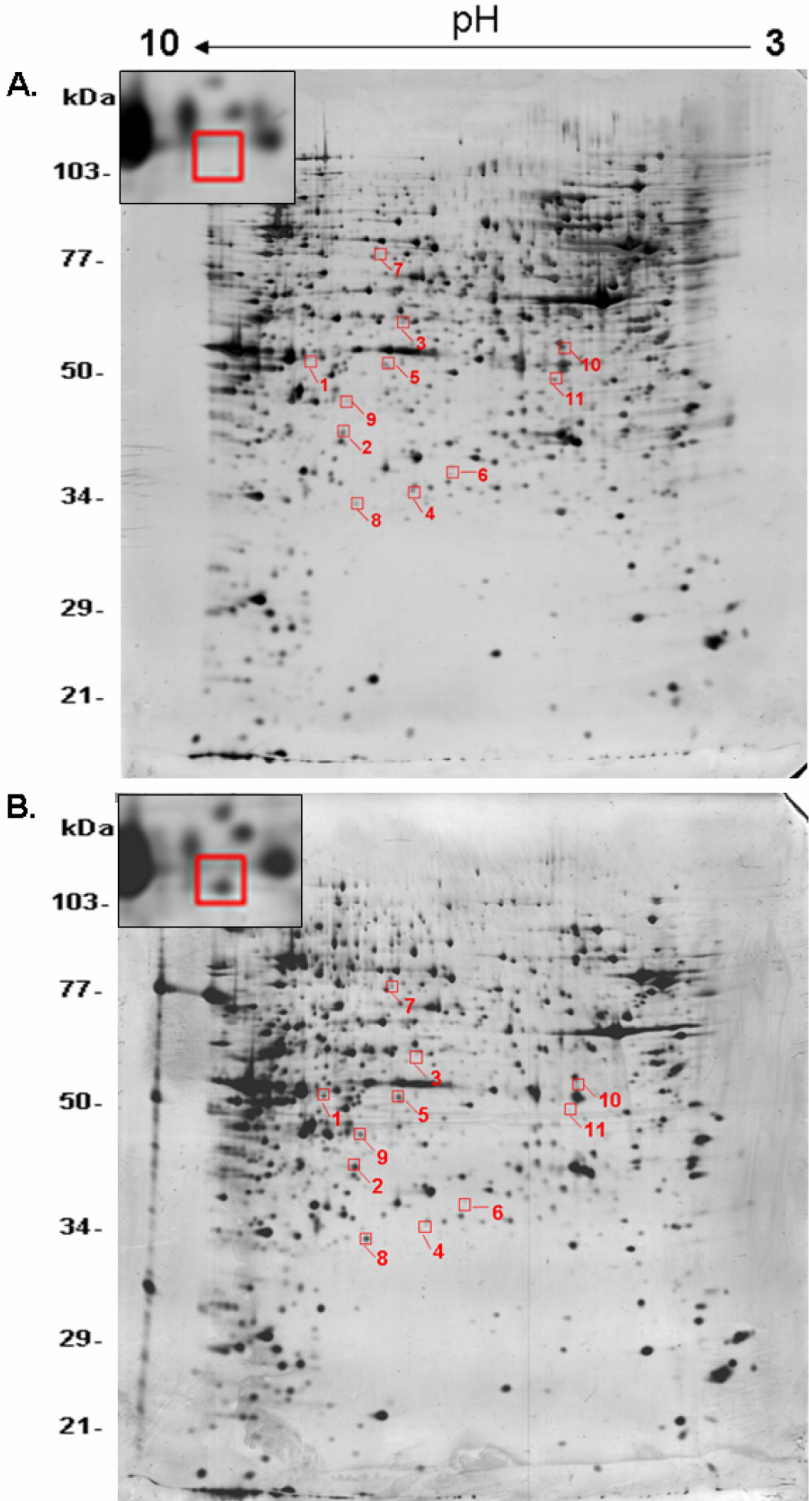
Two-dimensional electrophoresis comparison between NTM and GTM membrane protein extracts. Silver-stained 2D gels of membrane protein extracts from NTM (**A**) and GTM (**B**) are shown. Differentially abundant protein spots are highlighted in the red frames. Spot 1 was identified as copine1 (MW 58.6 kDa, pI 6.43). The expanded regions of differentially expressed copine1 were cut from the representative gels and pasted in the top left corner of the image. NTM: normal TM cells; GTM: glaucomatous TM cells.

Based on the comparison of two-dimensional electrophoresis (2-DE) patterns between membrane proteins of GTM and NTM cells, a total of 11 differentially abundant protein spots were found (Figure 2). Among them, five spots showed upregulation, and six spots showed down-regulation in GTM cell membranes. These protein spots were identified using a combination of peptide mass fingerprinting and MALDI-TOF analysis. Spot 1 was identified as copine1 (MW 58.6 kDa, pI 6.43). Spot 2 was FK506 binding protein 12-rapamycin associated protein (MW 48.7 kDa, pI 6.73). Spot 3 was ribosomal protein S6 kinase (MW 61.7 kDa, pI 6.42). Spot 4 was zinc finger protein 781 (MW 38.2 kDa, pI 6.38). Spot 5 was F22329_1 (MW 52.5 kDa, pI 6.24). Spot 6 was an unknown protein (MW 40.4 kDa, pI 5.78). Spot 7 was esophageal cancer-associated protein (MW 92.9 kDa, pI 6.38). Spot 8 was PSME3 (MW 29.5 kDa, pI 6.69). Spot 9 was Kenae2 (MW 44.2 kDa, pI 8.54) . Spot 10 was ANKRD15 protein (MW 84.1 kDa, pI 4.73). Spot 11 was S-arrestin (MW 45.1 kDa, pI 5.14). From the identified possible candidates, copine1 was selected for further analysis because of its clear and consistent abundance in GTM samples. As technical controls, all protein preparations and subsequent 2-DE analyses were repeated three times with a very high degree of resolution and reproducibility (data not shown).

### Upregulation of copine1 was verified in GTM cells but was not related to increased [Ca^2+^]i

**Figure 3:**
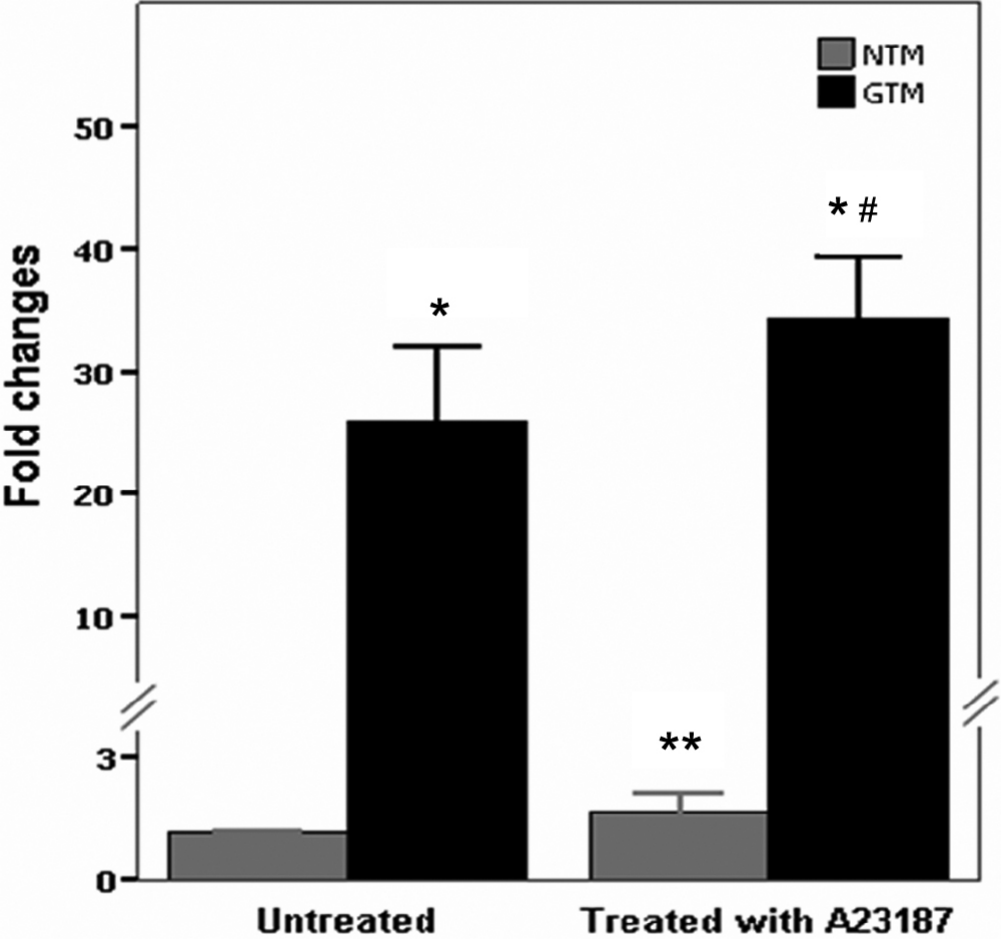
Confirmation of *copine1* mRNA expression in trabecular meshwork cells by real-time polymerase chain reaction using a relative quantification protocol. *Copine1* mRNA expression in untreated and A23187-treated TM cells was examined using real-time PCR. The comparative C_t_ method was employed, and the change in gene expression is expressed as fold change in relation to every control. For the A23187-treated cells, Ca^2+^ and A23187 were incubated with TM cells for 30 min before cell collection. NTM: normal TM cells; GTM: glaucomatous TM cells. Data are means±SEM of four independent experiments. The asterisk indicates a p<0.01 versus untreated NTM, the double asterisk indicated a p<0.01 versus untreated GTM, and the sharp (hash mark) indicates a p<0.01 versus NTM treated with A23187.

**Figure 4:**
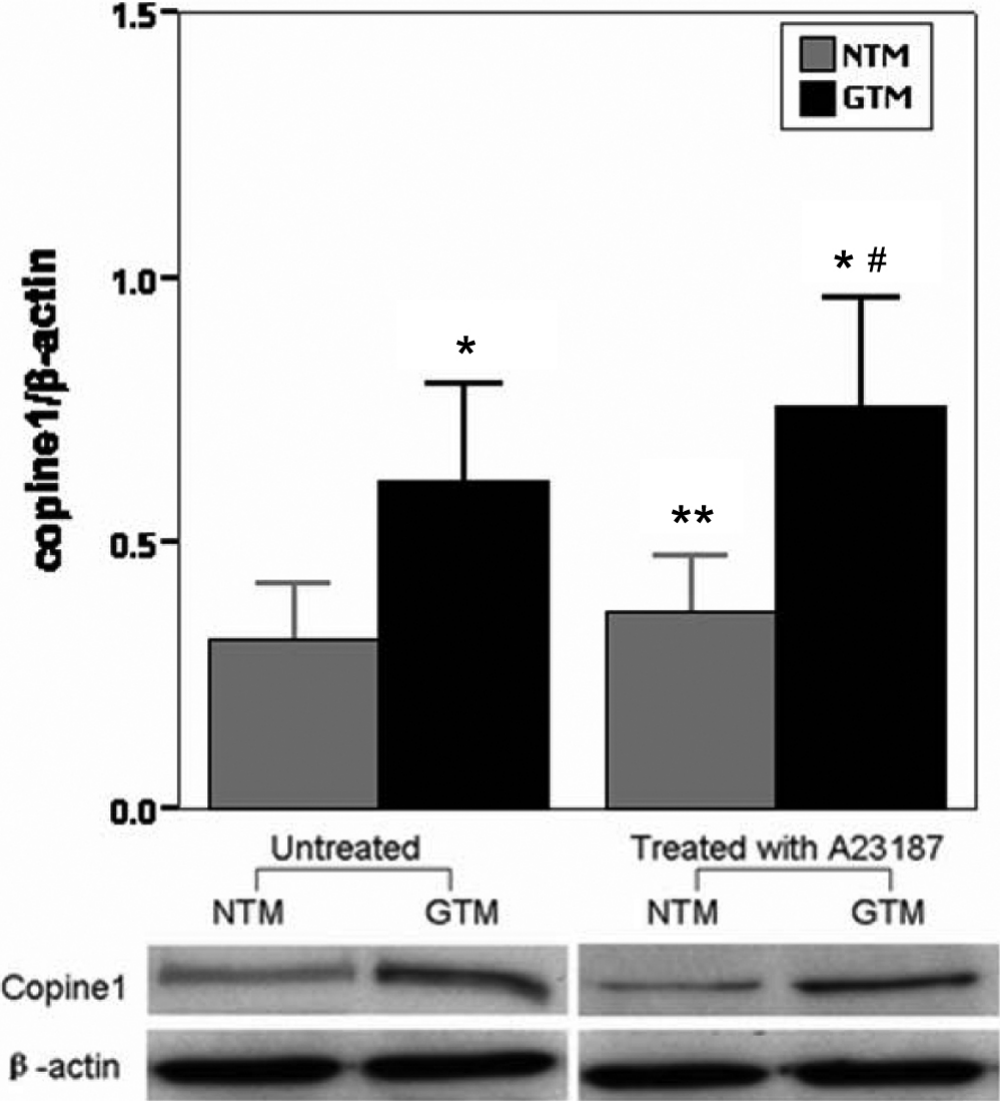
Western blot analysis using monoclonal antibodies against copine1 and β-actin on the traditional one-dimensional SDS–PAGE of trabecular meshwork cell total proteins. Expressions of untreated and A23187-treated copine1 were analyzed using the western blot method. The expression of β-actin was used as a control. The expression levels were quantitated by measuring band intensities and were expressed as fold induction of that in controls. NTM: normal TM cells; GTM: glaucomatous TM cells. Data are means±SEM of three independent experiments. The asterisk indicates a p<0.01 versus untreated NTM; the double asterisk indicates a p<0.01 versus untreated GTM; and the sharp (hash mark) indicates a p<0.01 versus NTM treated with A23187.

To verify the validity of 2-DE, we used relative quantification real-time PCR and the western blot method to quantify the expression of copine1 in NTM and GTM cells. The results were that copine1 was detected in both NTM and GTM cells; however, the mRNA and protein expression of copine1 in GTM cells increased 24.8±6.2 times and 1.8±0.3 times, respectively, when compared with those in NTM controls (Figure 3 and Figure 4).

Based on the interactions between calcium fluxes and the activity of copine1 [18,20,21], we further examined whether the influx of calcium could affect the expression of copine1. We introduced calcium into the TM cell lines by the use of a calcium ionophore, A23187, which can increase intracellular Ca^2+^ levels in intact cells. [Ca^2+^]i was measured by flow cytometry analysis using the fluorescent Ca^2+^ indicator, Fura 3 AM The scattergrams revealed that the application of A23187 markedly increased TM cell population in the Q2 region with high green fluorenscence (FITC-A) while there are only a few cells presented in the Q2 region in untreated controls. This result demonstrated that A23187 induced the influx of Ca^2+^ (data not shown). After the [Ca^2+^]i measurement, we detected effects of A23187 on the expression of copine1. Both the mRNA and protein expression results consistently showed no significant differences in copine1 expression between the A23187-treated TM cells and their untreated controls (Figure 3 and Figure 4). This indicated that the upregulation of copine1 in GTM cells was not related to increased [Ca^2+^]i.

### Copine1 displayed different distribution in GTM cells

**Figure 5:**
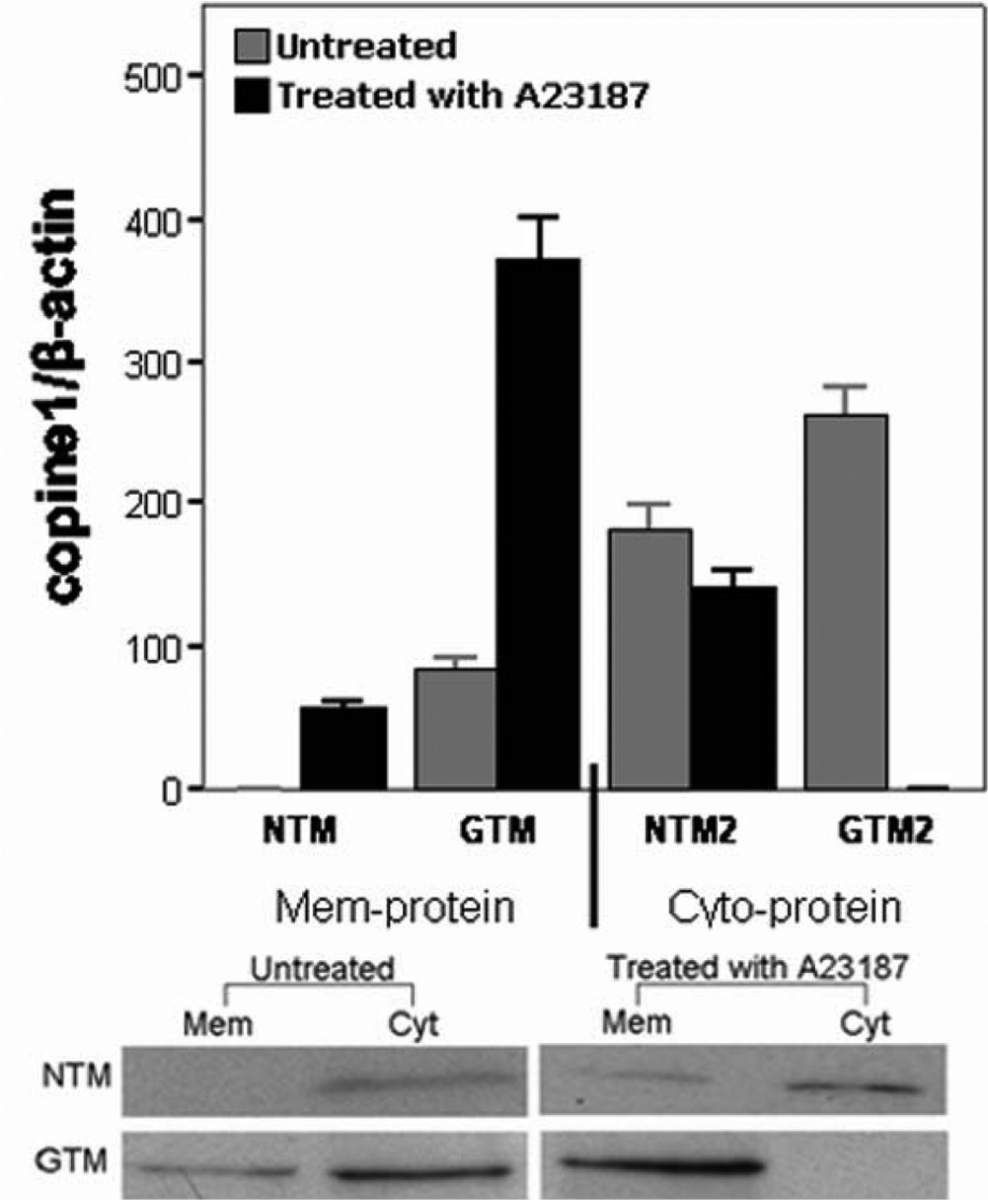
Subcellular distribution of copine1 and its calcium-dependent association with membrane. NTM and GTM cells either untreated or treated with A23187 were fractionated into a total membrane fraction (Mem) and a whole cell lysate (Cyt) as described in the Methods section. Following SDS–PAGE and western blot, copine1 was detected using a mouse monoclonal copine1 antibody. Equal amounts of protein were loaded in each lane, and the protein content was determined using the Bradford method (Bio-Rad) and BSA as the standard. For the A23187-treated cells, Ca^2+^ and A23187 were incubated with TM cells for 30 min before cell collection. NTM: normal TM cells; GTM: glaucomatous TM cells. Data from the three experiments were averaged and graphed with error bars representing standard errors.

We performed a cell distribution study of copine1 in TM cells as revealed by western blot probed with copine1 antibody. The membranes and cytoplasm of TM cells were fractionated as described above. An equal amount of each fraction (10 μg of protein) was analyzed using the western blot method. As shown in Figure 5, copine1 localized both in the cytoplasmic and membrane fractions of GTM cells. However, the immunoreactive band was only detected in the cytoplasmic fractions and not in the membrane fractions of NTM cells. These results suggested that copine1 in membrane fractions of GTM cells may be relative to the pathogenesis of POAG.

### Influx of Ca^2+^ redistributed copine1 completely to the membrane fraction in GTM cells

The distributional disparity of copine1 in GTM and NTM cells and its calcium-dependent property led us to investigate whether increased [Ca^2+^]i in TM cells altered the localization of copine1. To address this question, we determined the redistribution of copine1 in A23187-treated TM cells. The membrane and cytoplasm fractions were then analyzed using the western blot method using the copine1 antibody. In NTM cells, copine1 was detected mainly in the cytoplasm fractions, and only a trace of protein was detected in the membrane fractions. In contrast, copine1 was present mostly in the membrane fractions and was completely absent from the cytoplasm in GTM cells (Figure 5). These findings indicated that increased [Ca^2+^]i stimulated the translocation of copine1 from the cytoplasm to the membrane in GTM cells.

## Discussion

The biological and pharmacological importance of membrane proteins makes us presume that plasma membranes of TM cells change during the development of POAG. To characterize membrane proteins of TM cells potentially associated with POAG, we compared the membrane protein profiles of glaucomatous TM cells and normal controls. One finding of this study was that a membrane-associated protein, copine1, was largely upregulated in glaucomatous TM plasma membranes. Furthermore, its conspicuous Ca^2+^-dependent association with the membrane fraction was verified in glaucomatous TM cells using an in vitro fractionation procedure. Copine1 is a highly conserved membrane-binding protein found in a variety of eukaryotic organisms. Several studies have provided evidence that copine1 plays a role in growth control, exocytosis, apoptosis, gene transcription, and cytoskeletal organization and defense [18,22,23]. Although present in most major adult organs, copine1 has not previously been reported in ocular tissues. Therefore, the possibility that it plays a role in the pathogenesis of POAG has not been discussed.

Copine1 contains two Ca^2+^-dependent, phospholipid-binding domains (or C2 domains) at the NH_2_-terminal region, similar to those of protein kinase C and other proteins involved in the transduction of Ca^2+^ signals [24]. This suggests copine1 is involved in Ca^2+^-mediated intracellular processes. The most well documented example is that copine1 regulates TNF-α signaling through NF-κB in human embryonic kidney 293 cells in a Ca^2+^-dependant manner [18]. Although increased IOP in glaucoma is often associated with an influx of calcium, resulting in increased [Ca^2+^]i of TM cells [25-27], we found that increased [Ca^2+^]i could not increase the expression of copine1 in total cells. This indicates that upregulation of copine1 was not due to the increased level of [Ca^2+^]i in glaucomatous TM cells. However, in line with Tomsig’s recent findings in human embryonic kidney 293, human bladder carcinoma T24, and rat pheochromocytoma PC12 cells expressing human copine1 [18,28], we found that the influx of Ca^2+^ resulted in a complete redistribution of copine1 to membrane fractions in glaucomatous TM cells. These findings again demonstrate the characteristic Ca^2+^-dependent membrane translocation of copine1. Nevertheless, it is possible that the fractionation procedure of membrane and cytoplasm in TM cells, resulting in the change of actual calcium concentration, could affect the ability of copine1 to bind to phospholipids in the membrane fractions. This, in turn, may not reflect the distribution of copine1 in membrane fractions in the TM cells treated with A23187 and in the resting cells before fractionation procedure. Obviously, more work is needed to preclude this possibility, but at least some of the elements are in place. The membrane translocation of copine1 in this study at least in part explains the increased expression in plasma membranes of glaucomatous TM cells.

At the COOH-terminal region, copine1 has an “A domain,” which is similar in sequence to the von Willebrand A (VWA) domain [13,29]. VWA domains exist in several extracellular matrices (ECMs) and immune system components and interact with collagen proteins such as GpIbα and integrin αlibβ3. It has been suggested that they adhere to and cause the aggregation of platelets, macrophages, and TM cells [4,29,30]. TM is composed of a complex ECM of collagen beams lined with TM cells [31], and perturbation of the collagen or the ECM composition in TM may lead to the elevation of IOP and development of POAG as a result of impeded aqueous humor outflow [32]. It is well established that copine1 may be oligomerized with target proteins in a calcium-dependent manner and thereby activate the targets [33]. Indeed, it has been demonstrated that copine1 is able to recruit over 20 target proteins of which collagen is the only extracellular target identified by yeast two-hybrid screening [33]. Thus, we guess that copine1, just like cochlin in glaucomatous TM [4], may interact with fibrillar collagens through its VWA-like domains, although copine1 has not been demonstrated to have access to the outside of cells. Increased levels of copine1 may help to dissociate collagen from other TM ECM proteins, perturb collagen fibrillar assembly, trigger collagen degradation, and result in elevated IOP. These above-mentioned findings indicate that copine1 may, in a calcium-dependent manner, be an intriguing culprit in the pathogenesis of POAG.

Another interesting finding of this study is that only a slight translocation of copine1 occurred in normal TM cells in response to the influx of Ca^2+^ as a bulk translocation could have been expected. A possible reason for the discrepancy relates to the differences in the native expression of copine1 or protein activity between glaucomatous TM cells and normal controls. If the expression of copine1 is too low to bind the intracellular calcium, the target proteins are not transported to membranes, thereby causing a breakdown in the function of copine1. Thus, inactivated or low activation of copine1 in normal TM cells could not show a bulk response to Ca^2+^ influx, although this does not have to be the case.

In summary, we conclude that copine1 is upregulated in glaucomatous TM plasma membranes, and its upregulation may be explained partly by the Ca^2+^-dependent translocation to membrane fractions. However, the exact role copine1 plays in human glaucomatous TM cells still needs to be determined. Investigation of the role and functions of copine1 in TM cells should offer new insight into the abnormal intracellular Ca^2+^-signaling pathway in glaucomatous TM and help to clarify the molecular mechanism of POAG.
